# Decision making for breast cancer prevention among women at elevated risk

**DOI:** 10.1186/s13058-017-0826-5

**Published:** 2017-03-24

**Authors:** Tasleem J. Padamsee, Celia E. Wills, Lisa D. Yee, Electra D. Paskett

**Affiliations:** 1 Division of Health Services Management & Policy, College of Public Health, The Ohio State University, 280F Cunz Hall, 1841 Neil Avenue, Columbus, OH 43220 USA; 2 College of Nursing, The Ohio State University, Columbus, OH USA; 3 College of Medicine, The Ohio State University, Columbus, OH USA

## Abstract

Several medical management approaches have been shown to be effective in preventing breast cancer and detecting it early among women at elevated risk: 1) prophylactic mastectomy; 2) prophylactic oophorectomy; 3) chemoprevention; and 4) enhanced screening routines. To varying extents, however, these approaches are substantially underused relative to clinical practice recommendations. This article reviews the existing research on the uptake of these prevention approaches, the characteristics of women who are likely to use various methods, and the decision-making processes that underlie the differing choices of women. It also highlights important areas for future research, detailing the types of studies that are particularly needed in four key areas: documenting women’s perspectives on their own perceptions of risk and prevention decisions; explicit comparisons of available prevention pathways and their likely health effects; the psychological, interpersonal, and social processes of prevention decision making; and the dynamics of subgroup variation. Ultimately, this research could support the development of interventions that more fully empower women to make informed and values-consistent decisions, and to move towards favorable health outcomes.

## Background

Current risk estimation models enable the identification of women who are at elevated risk for breast cancer through genetic testing for *BRCA1*, *BRCA2*, and other mutations, as well as other potential genetic susceptibilities made evident by family histories of the disease. These high-risk women face a lifetime likelihood of breast cancer of between 20% and 80% depending on family history and genetic findings, significantly greater than the average 12% risk for women in the US. A subset of high-risk women face additional stresses related to genetic findings that do not correspond to a very specific risk estimate, or have unclear clinical implications, due to limitations of existing genetic science and risk quantification.

Several risk management options are available to support women at higher than average risk of breast cancer. Most of these are significantly underused by women who may benefit in terms of reduced cancer risk and cancer-related worry. Only about half of *BRCA* mutation carriers undergo the recommended prophylactic oophorectomy [1] and fewer than 5% of the high-risk women likely to benefit from chemoprevention use it [2, 3]. Underuse may be driven by multiple factors: lack of physician or patient information or understanding; lack of clinician confidence discussing preventive interventions or identifying women who could benefit from them; psychological or social dynamics that shape women’s preferences, deliberations, or ability to act on their decisions; and fully informed choice. Ultimately, it is women who make the choices—often with the help of health professionals and personal connections—about which prevention options to implement. These individual choices have significant impact on utilization of prevention options, breast cancer incidence, and health outcomes. Nevertheless, little is known about the processes women navigate as they make these decisions.

This article summarizes what is known and unknown about the various drivers of women’s decisions about breast cancer risk management methods. It begins with a brief overview of breast cancer prevention options for high-risk women, followed by a review of the current literature regarding decision making about these options by specific populations of women, and possible explanations for these patterns. This review also highlights important areas for future research, which could support the development of interventions that more fully empower women to make informed and values-consistent decisions and contribute to favorable health outcomes. It focuses solely on the prevention decision making of women at elevated risk of breast cancer due to identified genetic mutations or familial history. The prevention behavior of average risk women, decisions relevant after a breast cancer diagnosis, and the psychological sequelae of prevention interventions are outside the scope of this discussion.

## Breast cancer prevention pathways

Women at elevated risk for breast cancer are those who either have a known predisposing genetic mutation, or have a family history of breast or related cancers sufficient to raise calculated lifetime chance of breast cancer above a certain benchmark—usually 20–25% [4–13]. Studies of breast cancer patients and early population-based screening studies suggest that between 10% and 15% of women with a substantial family history likely carry *BRCA1/2* mutations [14, 15], and about half of *BRCA* mutation carriers are unaware of this status [16, 17]. Current evidence indicates that specific mutations in other genes (*ATM*, *CDH1*, *CHEK2*, *NBN*, *NF1*, *PALB2*, *PTEN*, *STK11*, and *TP53*) also confer increased breast cancer risk, and testing for these mutations is becoming increasingly available. Positive findings for these mutations are currently associated with recommendations to add magnetic resonance imaging (MRI) screenings and, for a subset of these genes, to consider prophylactic surgery, but other aspects of appropriate clinical management for these patients remain under investigation [13, 18–20]. Guidelines recommend that women with a family history of breast or related cancers be screened, receive genetic counseling and testing if indicated, and receive counseling to discuss chemoprevention, risk-reducing surgery, and enhanced surveillance options if found to meet familial or genetic risk criteria [13, 21, 22]. Four biomedical prevention options form the basis for women’s individual prevention pathways.

Bilateral prophylactic mastectomy (BPM; the surgical removal of both breasts for breast cancer risk reduction), the single most effective prevention method, reduces breast cancer risk by about 90% [23–31] and breast cancer-specific mortality by upwards of 80% [25, 26]. It may not improve overall survival, however, relative to routine mammography and MRI use, particularly for women who have had their ovaries removed [27]. Contralateral prophylactic mastectomy (CPM; surgical removal of the nonaffected breast for women with unilateral breast cancer) has not been shown to improve survival rates, but may decrease the risk of contralateral cancers in certain high-risk women; it is considered a clinically appropriate option for breast cancer patients with known *BRCA1/2* mutations, significant family history, or high-risk histology [32–38].

Prophylactic surgical removal of ovaries and fallopian tubes (bilateral prophylactic salpingo-oophorectomy; BPSO) is recommended for all *BRCA* mutation carriers between the ages of 35 and 40 years (or when childbearing is complete). For this group, it reduces the risk of ovarian, fallopian tube, or peritoneal cancer by 80% [39], likely halves the risk of breast cancer [33, 36, 40–43] (but see [44] for a counter-argument), and strongly reduces breast cancer mortality, ovarian cancer mortality, and all-cause mortality [27, 39]. However, adverse effects include induction of menopause, as well as increased risk of cardiovascular disease, osteoporosis, and cognitive impairment. Treatment with hormone replacement therapy (HRT) is controversial due to increased breast cancer risk [27, 45].

Two selective estrogen receptor modulators are approved for use as chemoprevention agents in the US. A 5-year course of tamoxifen by premenopausal women at elevated risk reduces their risk of breast cancer by 30% to 50%. Side effects include increased risk of endometrial cancer and venous thrombosis during the treatment period, while the protective benefits of chemoprevention last for at least 20 additional years [23, 46–52]. Raloxifene (approved for postmenopausal women) is estimated to be 76% as effective as tamoxifen in reducing the risk of invasive breast cancer, with significantly lower risks of thromboembolic events and uterine cancers [53–55]. Aromatase inhibitors show substantial promise as chemoprevention agents but are not yet approved for this use in the US or Europe; other potential chemoprevention agents including nonsteroidal anti-inflammatory drugs (NSAIDS), aspirin, metformin, cyclooxygenase-2 (COX-2) inhibitors, and poly-adenosine diphosphate-ribose polymerase (PARP) inhibitors have shown promise in early clinical research [27, 53, 56–59].

Enhanced surveillance is designed to facilitate early detection and treatment of breast cancer in women at high risk. Recommendations include: 1) increasing the frequency of clinical breast examinations to biannual checks; 2) initiation of radiologic screening at younger ages, such as 5 to 10 years prior to the youngest age of breast cancer diagnosis in a woman’s family; 3) annual bilateral screening mammograms, combined with targeted ultrasound examinations as indicated; and 4) the addition of breast MRI for women with a lifetime risk of breast cancer of 20% or greater [13, 60]. These methods substantially increase the probability of early cancer detection in high-risk women, but require sustained adherence, involve regular (and sometimes substantial) expenditures, and raise distress rates associated with false-positive tests [61–66].

Lifestyle changes that reduce the risk of breast cancer in the general population are considered wise but insufficient for those with higher, familial risk [30, 56, 67]. These include increased intake of vegetables, fruit, and fiber, increased exercise, weight management, smoking cessation, reductions in alcohol use, prolonged lactation, and minimizing exogenous hormone therapy [56, 68].

It is likely that high-risk women commonly compare the effectiveness and consequences of methods and consider particular combinations of prevention options. There is, however, a sparse evidence base for these comparisons and combinations [69, 70]. It is known that *BRCA* mutation carriers can achieve greater risk reduction by undergoing both BPM and BPSO than either alone [27, 71], and that prophylactic surgeries generally reduce both cancer risk and anxiety about cancer [28]. BPSO is likely the single intervention with the best risk-benefit ratio for *BRCA* mutation carriers [72, 73]. Prophylactic surgeries may be more cost-effective than other methods, but enhanced surveillance yields the most quality-adjusted life years [74, 75]. Given the serious ethical and practical impediments to randomized controlled trials, prospective observational studies that take into account adherence to chemoprevention and enhanced surveillance could be useful in comparing morbidity, mortality, and psychological consequences in the context of various prevention strategies across various subgroups of women [30, 76].

## Women’s prevention choices

### Uptake of prevention methods

Most research on uptake of biomedical prevention methods pertains specifically to *BRCA* mutation carriers, and less is known about women with apparent hereditary risk who are negative for *BRCA* mutations, those known to carry other risk-increasing genetic mutations, and those who have not undergone genetic testing. The rate of BPSO in *BRCA* mutation carriers ranges from 55% to 90% in various populations over periods ranging from 6 months to 10 years after receipt of genetic testing results [1, 77–81]. BPSO is performed on many additional women with hereditary risk each year, although the risk-reduction potential is less certain for women not known to have a *BRCA* mutation [42, 82]. Most studies find the rate of BPM among cancer-free *BRCA* mutation carriers to be about 20% and gradually rising [1, 77, 78, 83, 84], although it varies from as low as 11% to as high as 50% in specific samples [78, 81]. Overall, up to 80% of *BRCA* mutation carriers in some populations may undergo at least one risk-reducing surgery within 5 years of genetic testing [70], but this rate is likely much lower in other groups. BPSO is likely more common than BPM among *BRCA* mutation carriers because ovarian cancer treatment has poorer success rates than breast cancer treatment, because BPSO reduces risk of both ovarian and breast cancer, and because some women find mastectomy more psychologically difficult due to its potential effects on body image and sexuality [85]. CPM rates have risen substantially in recent decades, mostly among women who are *BRCA*-mutation negative or do not know their genetic status, and who are therefore unlikely to benefit [32, 38, 41, 43, 82, 86–89]. More than 5% of these women currently undergo CPM even as rates of contralateral breast cancer and regional breast cancer recurrence are both dropping; this raises concerns about surgery-related health risks, the need for new methods of communication about surgical options, and over-utilization of health services [42, 82, 89].

Population studies suggest that only 1–5% of women eligible to use tamoxifen for primary prevention actually do so [3, 67, 90–92]. Only 15% of *BRCA* mutation carriers approached for a chemoprevention trial enrolled [2], and only 8.5% of *BRCA* mutation carriers offered chemoprevention started such a regimen within 4 years of receiving genetic test results [77]. These usage rates fall far short of the proportion of women *interested* in chemoprevention, which one study found to be upwards of 40% [93] of those with familial risk. Chemoprevention also poses the challenge of long-term adherence: a large study of women at familial risk found that almost half of those who started tamoxifen chemoprevention did not complete the 5-year regimen [59, 94]. Additional studies are needed to fully understand the barriers to chemoprevention use, but they include: research gaps (limited risk prediction at the individual level and questions about risk-benefit profiles for specific subgroups); physician challenges (insufficient knowledge, difficulty identifying chemoprevention candidates, lack of training and confidence in risk assessment and counseling, and lack of time); and patient challenges (fear of side effects, predicted stress associated with chemoprevention, inaccurate or incomplete information, weighing witnessed experiences more heavily than statistical probabilities, and concerns about insurance coverage or cost) [3, 93–101]. It is important to understand low uptake better and to address the associated challenges, particularly in light of the recent review panel estimate that up to 50% of breast cancers among women at elevated risk could be prevented using currently available chemoprevention [56].

Between 20% and 50% of *BRCA* mutation carriers, and probably more high-risk women without known mutations, engage in surveillance alone, without specific intervention for biomedical risk reduction [70, 77]. Despite the widespread adoption of this ‘watchful waiting’ approach and recent increases in the use of screening MRI among women with familial risk [102], studies indicate that fewer than 70% of women are adherent to evidence-based screening recommendations, and that the use of screening varies by race and ethnicity [103–106]. Effective screening also requires access to accurate and personalized information about the appropriate screening schedule and to clinical, radiologic surveillance that is financially and logistically feasible.

The general relationship between lifestyle factors and breast cancer has been extensively studied, but the specific extent to which high-risk women use lifestyle changes to reduce breast cancer risk has not yet been explored. Future research should examine women’s perceptions and use of dietary and exercise changes as prevention behaviors, and how lifestyle choices relate to women’s other preventive decisions.

### Which women choose which prevention options?

Understanding which women are likely to make particular prevention choices is a key basis for efforts to facilitate informed, values-consistent, health-protective decisions. This involves understanding how uptake rates do or do not fit specific subgroups of women, as well as how psychological and social dynamics may affect the preferences and actions of individuals. Existing knowledge relevant to this area comes primarily from retrospective studies, usually of *BRCA1/2* mutation carriers and women who have completed preventive surgeries. This research is largely descriptive and correlational, and does not explore decision-making processes or other factors that affect women’s preferences and choices on a prospective basis.

#### Severity of risk, family history, and psychological health

Severity of cancer risk (both diagnosed and perceived) strongly affects prevention behavior. Higher perceived risk of breast cancer is positively associated with considering chemoprevention, BPM, and BPSO among high-risk women in general [83, 107, 108]. Known *BRCA* mutation carriers choose BPM more frequently than other women at elevated risk, and are more likely to believe that BPM is the best way to reduce both breast cancer risk and worry [109]. *BRCA* mutation carriers who believe ovarian cancer to be incurable are more likely to undergo BPSO [110]. Women with *BRCA1* and *BRCA2* mutations may behave differently with respect to BPM and BPSO, but this merits additional investigation [77, 111]. Among breast cancer patients, those who have clinical correlates of recurrence (larger tumors, lobular histology, known *BRCA 1/2* mutations) are in fact significantly more likely to choose CPM [111, 112]. The most influential correlates of CPM choice, however, are not these clinical factors but other patient factors: cancer worry, socioeconomic factors, and demographic factors [34, 89, 113].

Several studies point to strong but complex relationships between family history and surgical choice [114, 115]. *BRCA* mutation carriers with a first-degree family history of breast or ovarian cancer—particularly in a mother or sister—are more likely to undertake prophylactic surgery, and breast cancer patients with a family history are less likely to choose breast-conserving surgery and are more likely to undergo CPM [77, 108, 111]. Future research might clarify these relationships by disentangling two possible causes for the effects of family history on surgical choice: objective differences in cancer risk depending on a woman’s specific family history, and the personal impact of directly witnessing a close relative experiencing cancer.

Some evidence suggests that women’s psychological well-being may also affect their prevention choices. Among women with hereditary risk, BPM is more often chosen by those who experience high anxiety and/or exaggerated perceptions of their risk [116]. *BRCA* mutation carriers with poorer self-perceived health may be more likely to choose prophylactic surgery [110]. The choice to undergo CPM is also associated with psychological motivations, including higher cancer-specific distress, worries about recurrence or the efficacy of surveillance, and the concerns of significant others [34, 117–122].

#### Demographic characteristics

Existing research indicates that breast cancer morbidity and mortality, access to treatment, and decisions regarding a range of related screening, diagnostic, and treatment questions all differ substantially by race and ethnicity [103–106, 123–128]. It is thus likely that racial-ethnic variation also exists in the processes and outcomes of women’s prevention decision making. A few studies support this hypothesis, establishing that both BPM and CPM are most often chosen by white *BRCA* mutation carriers [82], and that African-Americans are less likely to participate in genetic risk assessment [129]. It would be helpful to know the extent to which use of BPSO and chemoprevention differ by race, how decision making and prevention choices vary among groups of non-white women, and which mechanisms (e.g., healthcare access, cultural differences, relationships to providers) underlie these racial-ethnic variations.

The significant body of research that relates socioeconomic status (SES) to healthcare access, general prevention behavior, and health outcomes suggests that cancer prevention decisions may also be systematically related to SES. However, the sparse research on this potential relationship has so far yielded conflicting findings on relationships between prevention-related choices and SES indicators including employment, education, and health insurance status [82, 130]. The lack of direct attention to relationships between SES and prevention decisions, and the prominence of attention to financial considerations in the work of patient advocacy organizations [131], also suggest that this is an area that merits further investigation. The influence of SES on decision making could operate through both direct mechanisms (e.g., women considering what they can afford) and indirect mechanisms (e.g., if SES influences the degree to which healthcare providers engage in shared decision making).

Prevention decisions also vary by age. Both BPM and CPM are associated with younger ages among *BRCA* mutation carriers [132], while the choice of BPSO is associated with older ages [1, 26, 109, 111, 133] (but see [81] for an exception). The effects of age on chemoprevention or enhanced surveillance behavior have not been well studied, but it is clear that decision making is particularly complex for young *BRCA* mutation carriers who are often single, childless, and not yet confident making life-altering decisions [134–137].

Prophylactic surgery is more often chosen by *BRCA* mutation carriers who have at least one child [112], and those with multiple children are even more likely to undergo BPM and/or BPSO [1, 26, 81, 109, 138]. Marital status could also affect prevention decisions, but this relationship has rarely been investigated [87].

Large geographic differences have been observed in the uptake of preventive surgeries, across both nations and subnational regions [81]. Uptake of chemoprevention and adherence to enhanced surveillance recommendations could vary geographically as well, and this should be investigated. The reasons for geographic variation are as yet unclear and also merit study; potential mechanisms include cultural differences, provider education or behavior, availability and policies relevant to specific risk management options within a healthcare system, and financial and geographic access to specialist healthcare and genetic testing.

## Women’s prevention decision making

### Information and communications

The acquisition and processing of accurate information are necessary conditions for appropriate decision making. Women’s physicians are a trusted but insufficiently studied source of information about cancer risk and prevention [139, 140]. Better informed patients make different decisions to others, and a substantial proportion of variation in the use of medical procedures can be attributed to a lack of solid information transfer from practitioner to patient, and lack of opportunities for patients to engage in shared medical decision making [45, 91, 141–147]. With respect to breast cancer prevention specifically, physicians vary in the provision of information and recommendations [148], often provide less information than high-risk women want [149, 150], frequently struggle to assess individual risk and eligibility for preventive procedures [67], and have difficulty navigating variations in patient preferences about the ideal degree of shared decision making [107, 148]. Intervention design research is warranted to improve physicians’ confidence in providing information [151], women’s confidence in making decisions based on that information, and the overall quality of information transfer. Future investigations should also examine whether confidence in risk information differentially affects women’s choices to pursue particular risk management behaviors.

For women with access to them, genetic counselors provide more thorough information about risk and prevention than generalist physicians or oncologists, combined with support to process information and make decisions [152]. Although the health information they provide can also vary [153], these interactions are associated with higher uptake of risk reduction methods [154]. Family, friends, patient communities, and survivor groups also have varied impact on women’s information gathering and processing [131, 148, 155], but additional research is needed about the conditions under which these relationships best support health-protective decision making.

### Decisional timing and complexity

For many women, prevention decisions are developed through complex processes that can involve explicit deliberation, objective information, intuitive and affective elements, and/or input from others [149, 156]. Perceptions of personal risk and prevention decisions evolve over a variable period of time [107, 140, 157–159]. Studies of *BRCA* mutation carriers who choose prophylactic surgery indicate that they may take several years to do so [63, 110], and that they are likely to make quicker decisions if they anticipate choosing surgery in the event of positive results before genetic testing, have first-degree relatives with cancer, experience higher psychological impacts of genetic findings, are older, have children, and/or experience specific triggering events [133, 160].

Qualitative studies have revealed that women’s conceptualizations of risk and prevention differ substantially from those of healthcare providers [161–163]. These distinctions reflect decision-making complexities far deeper than mere incomplete information or irrational decision making. The difficulty of prevention and surveillance choices for women at elevated risk likely reflects a range of normal cognitive patterns described by Daniel Kahneman, wherein intuitive and emotional decision making, shaped by personal experiences and instincts, is usually dominant over the conscious, analytic style of decision making. High-risk women report actively striving to make careful, deliberative breast cancer prevention decisions, but the cognitive dominance of the more reflexive decision-making mode may make this exceptionally difficult unless women have access to both thorough information and the cognitive skills necessary to process it [164]. Hesse-Biber and An further describe how prevention decision making in *BRCA* mutation carriers involves filtering genetic information through a complex framework of diverse psychological, social, and emotional factors [165]. Other research suggests some of the specific complexities that may shape women’s choices (nonlinear movement toward a decision [162]; acting to both maximize survival and preserve a sense of self [166, 167]; processing cancer experiences of primary relatives [157, 159, 168]; interpreting *BRCA* mutations as pressure to act [169]; and experiencing the uncertainties and interventions associated with elevated risk) are similar to those associated with breast cancer itself [96, 145, 163, 168, 170, 171]. These preliminary observations suggest that deeper attention to the meanings women construct around levels of risk, prevention options, diseases, and treatments may be an important element in understanding their decision making.

### Emotions

The stress associated with uncertainty is a central part of coping with health risks, and may be triggered at many stages of decision making [61, 148, 149]. Cancer-related worry or anxiety strongly motivates most women to take preventive action [109, 172–175]; particularly high levels of worry may also impede adherence to surveillance recommendations among some groups [176–178]. Some women are motivated to undertake preventive action through fear of abandoning their children, but others avoid surgeries that might cause their children to worry [140, 159]. Decisions can be changed or delayed by fear of surgery or side effects [67, 159]. Women’s decisions are also shaped by aspirations for their future (e.g., desire to have children), predictions about future emotions, perceived control over health, self-worth, experiences in high-risk families, risk fatigue, and cancer-related stigma [28, 97, 159, 164, 179]. Affective influences merit more thorough attention, and socio-emotional factors so far absent from the academic literature may also exert powerful influence on women’s decisions [164]. For instance, the broad body of research on patient-centered care, anecdotal news reports, and comments posted in online support communities all indicate that the desire to take control of one’s health can be a profound, but as-yet unstudied, part of the decision-making journey for women facing elevated risk of breast cancer [131, 180].

### Interactions among drivers of prevention choice

Finally, the impact of the drivers of decision making discussed above may not be consistent across subgroups of women, and a comprehensive understanding of interactive effects may be critical to the design of effective decision-support interventions. Relevant examples can be found in studies of race and ethnicity in decision making; for instance, subgroups of Asian-American women differ in their use of mammogram screening by ethnicity, health insurance, and SES [105, 181]. Minority populations also include a high proportion of the medically underserved [182], which suggests that understanding decision making may require attention not just to individual-level factors, but also to the communities in which women live and the resources they can access.

## Future research

Many of the most substantial gaps in the research literature have to do not with breast cancer risk reduction options themselves, but instead with how women make sense of and utilize these measures. Key gaps in existing knowledge of women’s prevention decision making are summarized in Table 1. Future research should focus on four key areas.

**Table 1:** Key gaps in current knowledge concerning breast cancer prevention decision making by women at elevated risk

• Which prevention options and combinations women consider viable (prevention pathways)
• Women's reasons for low uptake of biomedical prevention interventions
• Explicit comparisons of prevention options and their effects
• How prevention behavior varies among subgroups of women, who differ according to:
– Medically-defined or self-perceived level of risk
– Geographical and cultural context
– Race-ethnicity or socioeconomic status
– Access to medical information or care
• Mechanisms that account for variation in prevention choices across subgroups
• Effects of emotions and psychological factors on women’s prevention decision making
• Effects of spouses, children, family, and friends on decision making
• Effects of exposure to cancer patients, support groups, or advocacy organizations on decision making
• Effects of exposure to genetic counseling and quality of communication with other healthcare providers on decision making
• Effects of previously unstudied factors on decision making: stigma, self-worth, desire to take control of health, personal exposure to experience of cancer
• Interactions among various drivers of prevention choice
• How women at elevated risk explain their own decision-making processes and needs
• Key methods to help women attain informed and empowered decision making

### Women’s own perspectives

Prior research on women’s use of prevention interventions has been largely quantitative and deductive. This research has provided a powerful foundation for understanding women’s prevention behavior, but has also missed key dynamics such as the potential roles of financial resources, social support, and desire to control one’s health. The ability to support improved decision making will hinge on accurate understanding of women’s own perspectives, which can be illuminated by systematic qualitative research that offers women more space to articulate perspectives on their own experiences, rationales, and challenges.

### Explicit comparison of prevention pathways

The ability of women to compare their options would be facilitated by research that explicitly documents the effects of various choices on physical, psychological, and social well-being. One potential outcome of such research may be improved decision aids, which have been shown to positively impact knowledge, expectations, distress, and decisions among patients facing multiple medical options with complex pros and cons [183–189]. Initial steps have been made toward developing such tools for women at elevated risk (particular with respect to chemoprevention) [185, 190–200], but considerable work remains to incorporate all possible prevention pathways, and to consider psychological, social, and demographic factors in the construction of decision aids [97].

### Processes of decision making

Inductive research from women’s own perspectives may illuminate previously unstudied processes important to women’s decisions, and thereby offer new potential approaches for designing prevention-supportive interventions. At a minimum, these dynamics are likely to include: psychological factors from cancer-specific distress and fear for children to the desire to take control of health; social dynamics of support from spouses, family, and friends; exposure to cancer patients, survivors, and advocacy groups; the need to bolster both information acquisition and skills-building to facilitate deliberative decision making; and interactions among the drivers of prevention choice.

### Dynamics of subgroup variation

Existing research in and beyond the area of breast cancer prevention indicate that decision-making processes and prevention choices are likely influenced by the severity of medical risk, geographical context, race-ethnicity, SES, and access to medical information or care. These distinctions may have important implications for tailoring supportive interventions.

## Conclusions

One key conclusion of this review is that we must broaden our research agenda beyond the medical components of prevention interventions themselves, to focus also on social questions and women’s perspectives relevant to breast cancer prevention. Such research will help resolve crucial mismatches between the biomedical interventions researchers have developed to mitigate cancer risk and women’s real-life prevention behavior, and will ultimately provide critical support for the objective of preventing breast cancer among women at elevated risk.

## Abbreviations

ATM: Ataxia-telangiectasia mutated gene
BPM: Bilateral prophylactic mastectomy
BPSO: Bilateral prophylactic salpingo-oophorectomy
*BRCA1*: Breast cancer gene 1
*BRCA2*: Breast cancer gene 2
CDH1: Cadherin-1 gene
CHEK2: Checkpoint kinase 2 gene
COX-2: Cyclooxygenase-2
CPM: Contralateral prophylactic mastectomy
MRI: Magnetic resonance imaging
NBN: Nibrin coding gene
NF1: Neurofibrimin 1 gene
NSAIDS: Nonsteroidal anti-inflammatory drugs
PALB2: Partner and localizer of BRCA2 gene
PARP: Poly-adenosine diphosphate-ribose polymerase
PTEN: Phosphatase and tensin homolog gene
SES: Socioeconomic status
STK11: Serine/threonine kinase 11
TP53: Tumor protein p53
